# SSD-TSEFFM: New SSD Using Trident Feature and Squeeze and Extraction Feature Fusion

**DOI:** 10.3390/s20133630

**Published:** 2020-06-28

**Authors:** Young-Joon Hwang, Jin-Gu Lee, Un-Chul Moon, Ho-Hyun Park

**Affiliations:** School of Electrical and Electronics Engineering, Chung-Ang University, 84 Heukseok-ro, Dongjak-gu, Seoul 06974, Korea; mghysoka@cau.ac.kr (Y.-J.H.); dlwlsrn21@cau.ac.kr (J.-G.L.); ucmoon@cau.ac.kr (U.-C.M.)

**Keywords:** small-object detection, SSD, trident network, squeeze and excitation, feature fusion

## Abstract

The single shot multi-box detector (SSD) exhibits low accuracy in small-object detection; this is because it does not consider the scale contextual information between its layers, and the shallow layers lack adequate semantic information. To improve the accuracy of the original SSD, this paper proposes a new single shot multi-box detector using trident feature and squeeze and extraction feature fusion (SSD-TSEFFM); this detector employs the trident network and the squeeze and excitation feature fusion module. Furthermore, a trident feature module (TFM) is developed, inspired by the trident network, to consider the scale contextual information. The use of this module makes the proposed model robust to scale changes owing to the application of dilated convolution. Further, the squeeze and excitation block feature fusion module (SEFFM) is used to provide more semantic information to the model. The SSD-TSEFFM is compared with the faster regions with convolution neural network features (RCNN) (2015), SSD (2016), and DF-SSD (2020) on the PASCAL VOC 2007 and 2012 datasets. The experimental results demonstrate the high accuracy of the proposed model in small-object detection, in addition to a good overall accuracy. The SSD-TSEFFM achieved 80.4% mAP and 80.2% mAP on the 2007 and 2012 datasets, respectively. This indicates an average improvement of approximately 2% over other models.

## 1. Introduction

Object detection is an important area of computer vision with numerous applications in several fields such as autonomous driving [1], face detection [2], medical imaging [3], 3D reconstruction [4], optical character recognition [5], and action recognition [6]. Researchers have developed invariant feature extraction to more accurately identify objects; this has entailed challenges such as scale, rotation, viewpoint, and lighting variations. Traditional methods include histogram of oriented gradient (HOG) [7], SCALE invariant feature transform (SIFT) [6,8], and histogram equalization [9]. HOG is a dense feature extraction method, capable of extracting features for all locations in an image, as opposed to only extracting features for the local neighborhood of key points like the SIFT [8]. Ref. [2] reports the application of 3D-SIFT in action recognition. In Ref. [9], histogram equalization is employed as an approach for extracting features that are invariant to illumination and image capturing devices. These handcrafted feature methods are weak in generalization. However, the increase in graphics processing unit (GPU) power has led to numerous advances in deep learning architectures through the utilization of convolutional neural networks that extract more complex image characteristics.

Presently, object detection models that employ deep learning frameworks can be categorized into two-stage [10,11,12,13,14] and one-stage [15,16,17,18,19,20] models. In the two-stage models, region proposal and localization are performed sequentially. These models have large computational complexity, because the region proposal and detection tasks are performed separately. In contrast, the one-stage models perform these two tasks simultaneously, and consequently, these models are faster compared to their two-stage counterparts.

Among the one-stage models, the single shot multi-box detector (SSD) [16] has shown significant improvements in terms of accuracy. In Ref. [16], the SSD performed classification and localization of objects using anchor-boxes of different sizes at multiple scales by extracting different feature maps of various sizes. The SSD exhibited high performance in the detection of large objects, whereas small-object detection was conducted with low performance. Two reasons for this attribute are described in the following paragraphs.

First, the SSD does not consider the scale contextual information between its layers. Because feature maps are used independently as an input to predict the class and location, the scale contextual information between the layers is lost. The model also shows relatively low performance in the detection of small objects. To address this problem, a module, called the trident feature module (TFM), is proposed. This module is inspired by the trident network [21]. The TFM exploits and fuses feature maps at different dilation rates, thereby making the model robust to various scales.

Second, there is little semantic information contained in the shallow layers. This is because adequate semantic information is obtained by traversing several layers. Moreover, the amount of semantic information affects the final detection result. Shallow layers are specialized for detecting small objects via the extraction of feature maps with high resolution. However, as several layers are stacked in deep learning, the shallow layers tend to have fewer semantic properties. Consequently, deep learning-based SSD is not effective for small-object detection. The squeeze and extraction block and feature fusion module (SEFFM) is employed to compensate for the low amount of semantic information. The results extracted from the feature fusion module (FFM) are then used to focus on the useful feature maps in the squeeze and extraction (SE) block [22]. The SEFFM combines the feature maps obtained from deep as well as shallow layers. The performance of object detection can also be improved by reusing the feature maps. In this way, the semantic information of shallow layers can be effectively reinforced.

In this study, a modified SSD employing TFM and SEFFM, referred to as SSD-TSEFFM, is proposed. It is compared to the existing object detection models, such as the faster regions with convolution neural network features (RCNN) [12], SSD, and DF-SSD [20]. Experiments are conducted on two datasets: PASCAL VOC 2007 and PASCAL VOC 2012 [23]. The detection results are provided in the image form, and the accuracy results are provided numerically.

The main contributions of this study are as follows:

- A novel model with an accuracy higher than that of the SSD is proposed.
- TFM enhances the robustness of the model to feature maps with various scales in the proposed method.
- The proposed model, SSD-TSEFFM, addresses the challenges encountered in the detection of small objects.
- To evaluate the performance of the proposed model, SSD-TSEFFM is compared with the faster RCNN [12], SSD, and DF-SSD [20].

The remainder of this paper is organized as follows. Section 2 describes the related studies. Section 3 describes the proposed model, SSD-TSEFFM, in detail. Section 4 reports the experimental results and compares them with those of other models. The conclusions are drawn in Section 5.

## 2. Related Work

### 2.1. SSD Series

Before the introduction of the SSD [16], you only look once (YOLO) [15] was widely used. YOLO divides the image into grids and transforms these grids into regression targets for estimating the bounding boxes and class probabilities. However, this model suffers from low accuracy. This is because the YOLO utilizes only the last layer, which has coarse features. Unlike YOLO, SSD uses feature maps extracted from several layers.

**Figure 1:**
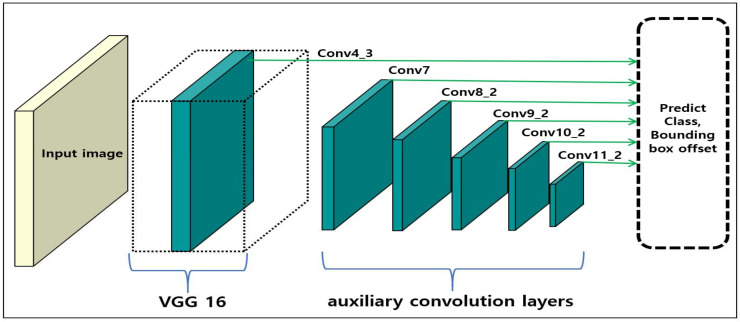
SSD—multiple feature maps (Conv4_3, Conv7, Conv8_2, Conv9_2, Conv10_2, and Conv11_2) are used to independently predict the class and location of the objects.

Figure 1 illustrates a basic SSD model consisting of a visual geometry group (VGG) [24] and auxiliary convolution layers. Object class and location are predicted independently using feature maps from each layer. Then, candidate groups of the predicted class and location boxes are reduced via non-maximum suppression. By using feature maps of each layer, the SSD shows improved accuracy in comparison to the existing methods. However, challenges are encountered in the detection of small objects, as contextual information is not considered.

The deconvolutional single shot detector (DSSD) [18] adds extra deconvolution layers at the end of the SSD. By integrating every prediction layer with its corresponding deconvolution layer, the contextual information can be injected into shallow layers, which leads to an improvement in the accuracy of small-object detection, as the resolution of the feature maps is enhanced. However, the use of the DSSD leads to computational complexity because the DSSD performs additional tasks owing to the addition of the extra layers.

The rainbow single shot detector (RSSD) [19] was developed to allow better predictions, by combining feature maps, than the ones made by SSD. Furthermore, SSD has low performance, as it does not reuse feature maps. In contrast, the RSSD showed improvements in accuracy by employing rainbow concatenation, where pooling and deconvolution operations are performed on a formed feature pyramid. However, applying significant concatenation increases the number of channels and the computation.

### 2.2. Object Detectors for Scale-Variance

Obtaining characteristics of objects at various scales is a crucial task for object detectors in the detection of small objects. To address scale imbalance, the scale normalization for image pyramids (SNIP) [25] performs scale normalization by applying the image pyramid [26] during the training and detection phases. Therefore, SNIP backpropagates only the loss of the selected scale. However, it has the disadvantage of a long computation time required by the pyramid method.

The scale normalization for image pyramids with efficient resampling (SNIPER) [27] improves upon the SNIP method by processing only the contextual region around the ground truth. This significantly speeds up the multi-scale training process, as SNIPER operates on low-resolution image patches. Owing to its memory-efficient design, SNIPER benefits from batch normalization during training and makes the construction of larger batch-sizes possible for instance-level recognition tasks on a single GPU. However, SNIPER crops the object into multiple parts, which reduces detection accuracy.

The trident network [21] resolves the scale variation problem by introducing multiple-scale features using dilated convolution. This network employs dilated convolution [28] to enable the use of feature maps at various scales. Feature maps can be extracted in parallel using different dilation rates and then synthesized to obtain feature maps that are robust to scale changes. This network utilizes a weight-sharing method that enables the rapid inference of objects without the need of several parameters and additional computing cost.

### 2.3. Feature Pyramid Network

In the image pyramid method [26], images are integrated by progressively reducing image scales. The features consequently become progressively smaller and accumulate, similar to a pyramid. Because this network synthesizes and utilizes features of different scales at each layer, it is robust to scale changes. However, since the image pyramid is infeasible in terms of memory, the use of this method can slow down the entire network.

In the feature pyramid network (FPN) [14], the feature map is used instead of the image itself. This proves to be relatively faster in terms of inference time, because it involves fewer computing operations as compared to the image pyramid. Furthermore, the FPN fuses feature maps from different layers, thereby leading to performance improvement in terms of accuracy. Although the FPN is faster than the image pyramid network, it can slow down the process of operating in two stages and receiving information in a serial manner.

### 2.4. Squeeze and Excitation (SE) Block

The SE block [22] involves two processes, namely the squeeze and excitation operations. The squeeze operation is used to extract important features from the channels. The important features are concentrated through global average pooling (GAP), and the remainder is excluded. Subsequently, an excitation operation is used to scale the importance of values of the feature map channels between zero and one. Through this process, the values of the feature maps are normalized, such that the maps can be easily synthesized. Furthermore, in this process, both the model and the computational complexity do not increase drastically. Therefore, although there is an increase in the number of parameters, the improvement in performance is significant. Furthermore, the SE block can also be used in this method for feature fusion.

## 3. Proposed Model

**Figure 2:**
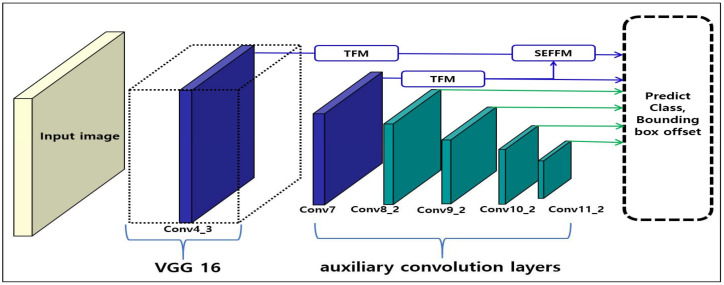
Overall structure of the single shot multi-box detector using trident feature and squeeze and extraction feature fusion (SSD-TSEFFM)—Conv4_3 and Conv7 feature maps pass through the trident feature module (TFM) and the squeeze and excitation block feature fusion module (SEFFM) to form new feature maps.

Figure 2 depicts the overall structure of the proposed SSD-TSEFFM. In VGG [24], the features are extracted and sent to the subsequent layers. Then, these features are used to predict the location and class of the objects in auxiliary convolution layers. In SSD [16], when detecting the location and class of objects, each layer is utilized independently. However, feature maps with varying scales are difficult to use, and initial shallow layers lack semantic information; these problems may lower the accuracy of small-object detection. Unlike the SSD, the proposed model employs two novel modules, namely the TFM and the SEFFM. The TFM is used to pass input feature maps into three different receptive fields through a dilated convolution [28]. This module addresses the problem of scale variability of the feature maps. The SEFFM is used to fuse the feature maps between the initial shallow layers and deeper layers. Consequently, the problem of little semantic information in the shallow layers is addressed.

### 3.1. Trident Feature Module (TFM)

**Figure 3:**
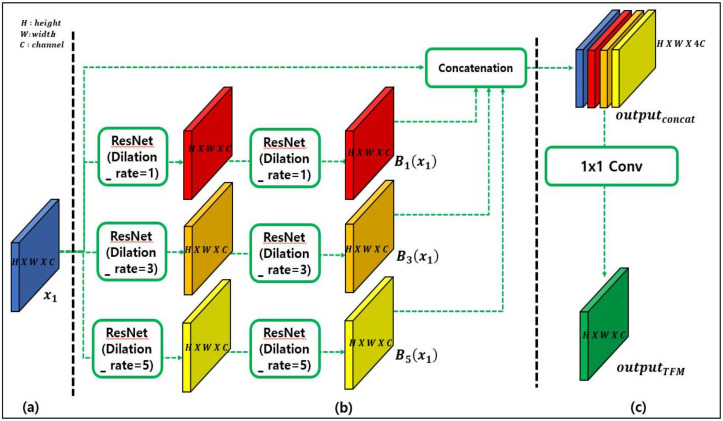
Trident feature module (TFM) structure illustrating three constituent parts. Part (**a**) presents the input feature map. Part (**b**) includes ResNet blocks with different dilation rates and concatenation. Part (**c**) illustrates the process of deriving the output.

In deep learning-based object detection models, the size of the feature maps is reduced using pooling layers. This is executed to remove some of the noise and extract important features. However, while reducing the feature maps, relatively small objects may be perceived as noise and disregarded. This problem is addressed by supplementing the scale information to the feature maps. Dilated convolution can be used to obtain contextual information on scale diversity, which allows small-object features to be recognized as important features. The TFM can be used to extract a feature map at three dilation rates, as depicted in Figure 3. This module was inspired by Ref. [21].

Figure 3a depicts the input feature map for TFM. The use of TFM is recommended in shallow layers, as the features of small objects disappear in deep layers. Therefore, the feature maps of Conv4_3 and Conv7, as shown in Figure 2, are used as inputs to the TFM. This process is described in detail in Section 4.3.

In Figure 3b, the input feature map is divided into three dilation rates of 1, 3, and 5 and processed in parallel with each residual network (ResNet) [29] block. Notably, only the dilation rates are different, while other parameters remain unchanged. Therefore, the computation of all ResNet blocks proceeds in a simultaneous manner. Feature maps extracted at the three dilation rates are concatenated with the original input feature map as follows:

(1) $$B_{dr}\left(x_{t}\right)=ResNet_{dr}\left(x_{t};dr\right)\;Output_{concat}=Concat\left(x_{1},B_{1}\left(x_{1}\right),B_{3}\left(x_{1}\right),\;B_{5}\left(x_{1}\right)\right),$$

where *dr* denotes the dilation rate, and $x_{t}$ represents the *t*-th feature map. For example, if $x_{1}$ refers to the first input feature map, then $x_{t}\in {\mathbb{R}}^{H\times W\times C}$. H, W, and C denote the height, weight, and channel size, respectively. When the three blocks and initial input values are concatenated, the dimension of the $Output_{concat}$ becomes ${\mathbb{R}}^{H\times W\times 4C}$**.** Through this process, it becomes possible for the feature to have both input as well as scale contextual information.

Figure 3c depicts the process of merging the channels. Through a 1×1 convolution, the channels are merged, such that the number of channels becomes the same as that of the input feature map:

(2) $$Output_{TFM}=Conv_{1\times 1}\left(Output_{concat},\;C\right),$$

where $Conv_{1\times 1}\left(\ldots \right)$ is a 1×1 convolution, and C represents the channel size. The result is projected in the channel size and the dimension, $Output_{TFM}\in {\mathbb{R}}^{H\times W\times C}$. Consequently, after passing through the TFM, the size of the feature map does not change, but only the scale contextual information is injected into the feature map.

### 3.2. SE-Feature Fusion Module (SEFFM)

Because the model determines the classes and locations of the objects at the end, the amount of semantic information increases as the layers of the model become deeper. Therefore, shallow layers have relatively less semantic information than deep layers. Hence, the amount of semantic information of small objects, which are mainly determined from the shallow layers, has little effect on the detection results. This issue can be addressed by reusing the feature maps in the shallow layers. SEFFM is used to synthesize the feature maps of both shallow and deeper layers.

**Figure 4:**
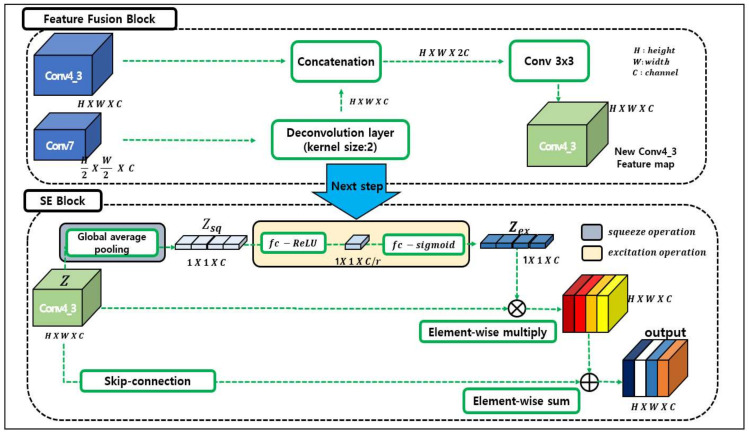
Feature fusion block and squeeze and excitation (SE) block. SE-feature fusion module consists of the SE-block and feature fusion block. The feature fusion block adds a feature map through the deconvolution layer (**upper** panel). The SE block compresses the channel and performs the operation to obtain weights of scale-related channels (**lower** panel).

As shown in Figure 4, the SEFFM consists of an FFM and a SE block [22]. The FFM is used to synthesize different feature maps of the same size, and the SE block is used to squeeze out key information. The size of the shallow feature map, Conv4_3, differs from that of the deeper feature map, Conv7. To make the sizes of the feature maps equivalent, deconvolution is performed on Conv7. The generated map undergoes concatenation and Conv 1×1 operation. The newly produced feature map, Conv4_3, is transferred to the SE block.

The SE block performs the function of an attention mechanism. In this block, the network concentrates on the useful features obtained from global information. Thus, the useful features are squeezed out. The input feature is expressed as $Z\left(Z\in {\mathbb{R}}^{C\times H\times W}\right)$. Further, each element in the input feature is expressed as $Z_{c}\left(Z_{c}\in {\mathbb{R}}^{H\times W}\right)$. The squeeze operation can be expressed as in Ref. [22]:

(3) $$Z_{\mathrm{sq}}=F_{\mathrm{sq}}\left(Z_{c}\right)=\frac{1}{W\times H}\overset{W}{\underset{i=1}{\sum }}\overset{H}{\underset{j=1}{\sum }}Z_{c}\left(i,j\right),\;\mathrm{for}\;c=\left(1,2,3,\ldots ,C\right),$$

where $Z_{\mathrm{sq}}$ is a squeeze feature map, and W and H are the width and height of the input feature map, respectively. $F_{\mathrm{sq}}$ refers to GAP; C refers to the size of the channel. Each $Z_{c}$ is converted to a 1 × 1 size scalar through global average pooling (GAP). That is, $Z_{\mathrm{sq}}$ is a feature map of the size, 1×1×C.

Then, the adaptive recalibration process is performed to select meaningful features from the ones that were squeezed out. This process is called excitation. The excitation operation can be expressed by Equation (4) as follows:

(4) $$Z_{\mathrm{ex}}=F_{ex}\left(Z_{sq}\;,\;W\right)=\sigma \left(W_{2}\delta \left(W_{1}Z_{sq}\right)\right),\;where\;W_{1}\in {\mathbb{R}}^{\frac{C}{r}\times C},\;W_{2}\in {\mathbb{R}}^{C\times \frac{C}{r}},$$

where $Z_{\mathrm{ex}}$ is the result of the excitation operation, σ is a sigmoid function, δ is a ReLU function, and $W_{1}\;\mathrm{and}\;W_{2}\;$represent fully connected layers. The output $\left(W_{2}\delta \left(W_{1}Z_{sq}\right)\right)$ is activated by σ, a sigmoid function, and therefore, it has a value between 0 and 1. $Z_{ex}$ identifies the relative importance of each channel as values of 0 and 1. After the excitation operation is complete, the module enhances $Z_{\mathrm{ex}}$ and input feature map Z by element-wise multiplication. Finally, the new map, Conv4_3, and the output of the SE block are combined through element-wise summation. The output thus obtained is focused on important features.

## 4. Experiment

### 4.1. Training Setting

Most of the training strategies employed in this study, including loss functions and data augmentation, followed the ones presented in Ref. [16]. The hard negative mining technique is adopted, such that the ratio between the positive and negative samples is at most 3:1, which helps ensure fast optimization and stable training. If the intersection-over-union (IOU) is 0.5 higher than the ground truth, a positive match is determined.

The proposed model was trained on the PASCAL VOC 2007, 2012 training and validation datasets (trainval) [23]. The learning rate was set to $10^{-3}$ in the first 80,000 iterations, $10^{-4}$ in the next 100,000 iterations, and for the remaining iterations, it was set to $10^{-5}$. Then, the entire network was fine-tuned using the SGD algorithm with 0.9 momentum and 0.0005 weight decay. A backbone network, VGG, was pretrained on the ILSVRC CLS-LOC dataset [30], wherein the weights of previous layers were retained to significantly shorten the training time. The newly added layers were initialized using the Xavier [31] method to allow the gradient values to maintain approximately the same proportion at each layer of the network. For a 300 × 300 input, the batch size was 16. For a 512 × 512 input, the model was tested, and the batch size was set to 8 by considering the GPU specifications. In.the TFM, dilation rates were set to 1, 3, and 5.

The proposed model was also evaluated using the training dataset, PASCAL VOC 2012, which contained a total of 21,503 images of VOC 2007 trainval + test (9963) and VOC2012 trainval (11,540). Because there were more data in this training dataset, the number of training iterations was increased. A total of 150,000 iterations were performed. The learning rate was $10^{-3}$ for the first 60,000 iterations, $10^{-4}$ for the next 120,000 iterations, and for the remaining iterations, it was set to $10^{-5}$.

The proposed model was tested on PASCAL VOC 2007, 2012 datasets, each containing 20 classes. The model detection performance was evaluated with regard to the mean average precision (mAP). SSD-TSEFFM was implemented using the Pytorch framework [32] and cuDNN v5.1 [33]. The hardware environment was an Intel Xeon E5-2620V, Nvidia-2080ti GPU.

### 4.2. Experiment Results

▪ Results on PASCAL VOC 2007 dataset

Table 1 presents the results of the SSD-TSEFFM on PASCAL VOC 2007. The proposed model was tested with input images of 300 × 300 and 512 × 512 size, respectively. When the size of the input was 300 × 300, SSD-TSEFFM achieved 78.6% mAP, which is 1.4% points higher than the original SSD300 and 2.2% points higher than the faster RCNN. When the size of the input image was increased to 512 × 512, the proposed model performed better by 0.9% points as compared to SSD512. Compared to other detectors, SSD-TSEFFM performed better on 13 out of 20 objects for inputs with a size of 300 × 300 and on 17 out of 20 objects for inputs with a size of 512 × 512.

The proposed model achieves more than 80% accuracy in the case of objects such as a bike, bus, car, mbike, etc. In contrast, for objects such as a bottle, chair, and plants, an accuracy of 60% or less is obtained. If the entire object is not visible because of cropping or occlusion, it is difficult for the detector to predict the object. Therefore, the detector shows low accuracy when there is only part of the object in the image, such as bottlenecks, chair backs, and leaves. High-accuracy results are efficiently obtained when all parts of the objects, such as wheels and bodywork, are visible. Because the SSD-TSEFFM is a model that exhibits improved performance when an object is small, it appears to perform better in a situation where the entire object can be identified.

▪ Results on PASCAL VOC 2012 dataset

The results on PASCAL VOC 2012 are presented in Table 2. The proposed model is also tested with input images with sizes of 300 × 300 and 512 × 512. The SSD-TSEFFM300 achieved 77.1% mAP, which is 1.3% points higher than that of the original SSD300. The accuracy of the SSD-TSEFFM300 was 3.3% points higher than that of the faster RCNN and 0.6% points higher than that of the DF-SSD300. SSD-TSEFFM shows an improvement with regard to testing tasks with specific backgrounds, and examples are bus (84.0% mAP), cow (81.9% mAP), motorbike (88.8% mAP), etc. Moreover, SSD-TSEFFM exhibits performance that is 0.5% and 2.4% points higher than that of SSD300 in the detection of small objects such as chairs and plants, respectively. The SSD-TSEFFM512 also showed a good performance, with 80.2% mAP, which is 1.7% points higher than that of the SSD512 model.

**Figure 5:**
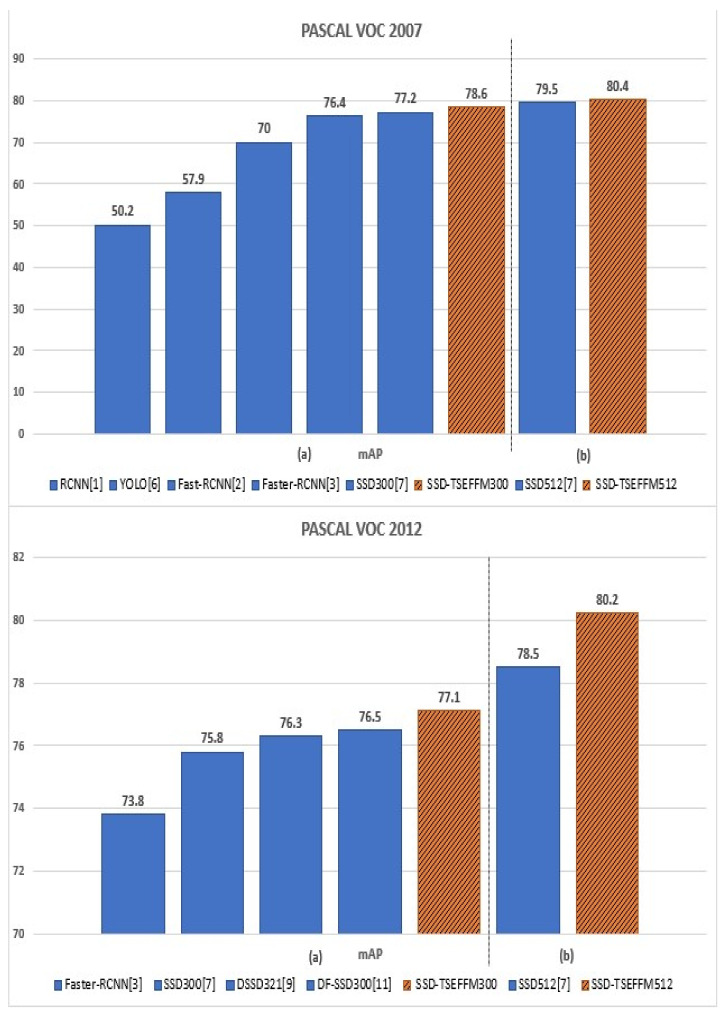
Diagram of mean average precision results tested on PASCAL VOC 2007 (**upper** panel), 2012 (**lower** panel) datasets. (**a**) depicts the result obtained with the input size of 300 × 300, and (**b**) shows the result of experiments with the input size of 512 × 512.

The comparison of mAP results is shown in Figure 5. The results are plotted for PASCAL VOC 2007 and 2012 datasets, and the mAP increases gradually from left to right for both. The results of the proposed model are shown in orange with diagonal hatched lines, whereas those of other models are shown in blue.

**Figure 6:**
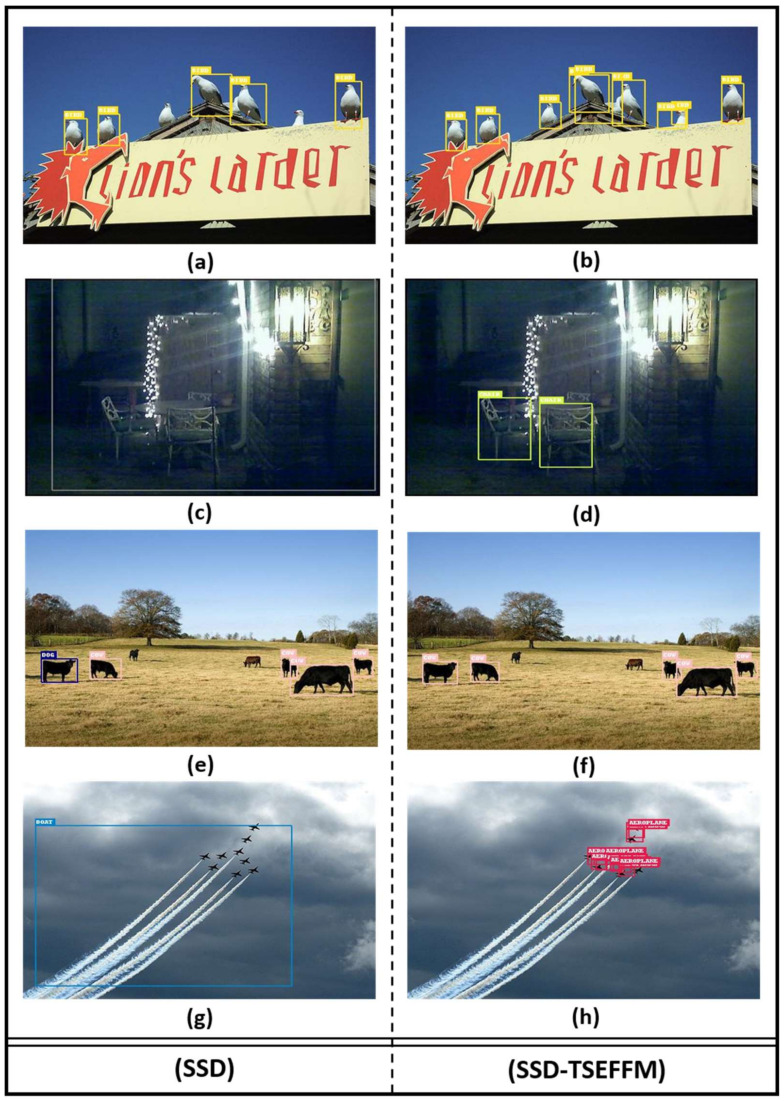

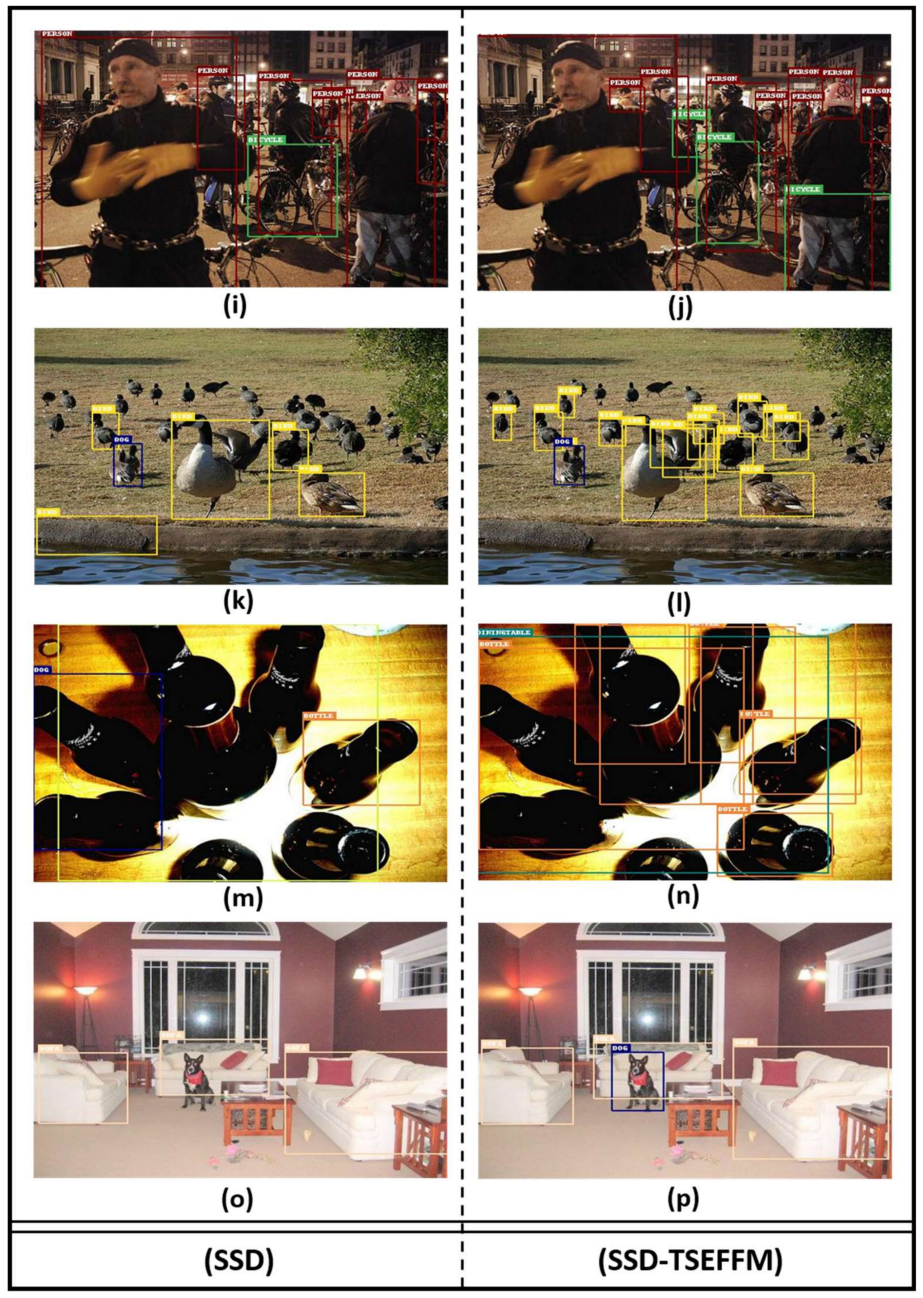
Comparison of object detection results of SSD-TSEFFM and SSD models tested on Pascal VOC dataset. **Left** panel images (**a**,**c**,**e**,**g**,**i**,**k**,**m**,**o**) are results obtained by SSD; **right** panel images (**b**,**d**,**f**,**h**,**j**,**l**,**n**,**p**) are results obtained by SSD-TSEFFM.

Figure 6 shows that the SSD-TSEFFM finds small objects more efficiently than SSD, where the left panel (a, c, e, g, i, k, m, o) shows the results obtained for SSD, and the right panel (b, d, f, h, j, l, n, p) shows those obtained for SSD-TSEFFM. For example, the dog marked by the blue label in Figure 6c depicts an incorrect result detected by SSD, whereas the cow marked by the pink label on Figure 6d depicts a correct result detected by the proposed model. Further, the dog in Figure 6o is not labeled by SSD, whereas it is detected by SSD-TSEFFM (Figure 6p).

### 4.3. TFM Application Results

To demonstrate the effectiveness of the TFM and determine which layer needs to be applied to the module, the PASCAL VOC 2007 dataset was employed. Based on the results presented in Table 3, adding the TFM leads to an improvement in the accuracy in small-object detection. The proposed model therefore shows a better mAP than the original SSD.

When applied to layers, from Conv4_3 to Conv8_2 or Conv9_2, the model yielded the highest mAP value, i.e., 78.7% mAP, which is 1.5% points more than that of the original SSD. However, the largest increase in mAP was observed when the TFM was applied to Conv7. With regard to the cases where TFM was applied to Conv10_2 and Conv11_2, the mAP values were observed to decrease. Further, as more modules are applied, the models become heavier, such as in the case of Conv8_2 and Conv9_2 SSD-TSEFFM models, as compared to the Conv7 SSD-TSEFFM model. Therefore, the most optimal model is the one where the modules are applied up to Conv7.

## 5. Conclusions

The SSD model [16] can independently detect objects using a single layer. However, because the shallow layers in this model lack semantic information, the accuracy of small-object detection is poor. The proposed model, SSD-TSEFFM, addresses this problem by the use of two modules, TFM and SEFFM. In TFM, the feature maps learn additional receptive fields, thereby making the model robust to feature maps of various scales. In SEFFM, feature maps extracted from different layers are synthesized, and because the feature maps have different sizes, scaling is necessary. Using the two aforementioned modules, the information in the shallow layers is enhanced, and the accuracy of small-object detection is increased. To evaluate the performance of the proposed model, the SSD-TSEFFM was compared with the fast-RCNN [12], SSD, and DF-SSD, using the same datasets [20]. Based on these results, an improved mAP was confirmed on the Pascal VOC 2007 and 2012 datasets [23], and the small-object search was confirmed based on the visual experiment results. Further, the SSD-TSEFFM demonstrated improvement in small-object detection and the overall accuracy by properly utilizing the shallow layer of the feature map.

However, the SSD-TSEFFM is deficient in capturing objects when parts of them are not visible owing to cropping or occlusion. This is because the proposed model has the limitation of adding information only to shallow layers. Deep layers have a high semantic value but low resolution, leading to difficulties in detecting parts of objects. Therefore, it is expected that increasing the resolution while preserving the high semantic value will improve the model. Furthermore, by appropriately modifying the proposed model, it may also be possible to propose a new model rather than a module synthesis. Therefore, in future work, the resolution of the deep layers must be improved, and further work must be conducted to develop a better model by including the module operations in the model itself.

## Figures and Tables

**Table 1:** Average precision results tested on Pascal VOC 2007 dataset (Bold values indicate the maxima).

Network	mAP	Aero	Bike	Bird	Boat	Bottle	Bus	Car	Cat	Chair	Cow	Table	Dog	Horse	Mbike	Persn	Plant	Sheep	Sofa	Train	tv
300 × 300 input																					
RCNN [5]	50.2	67.1	64.1	46.7	32.0	30.5	56.4	57.2	65.9	27.0	47.3	40.9	66.6	57.8	65.9	53.6	26.7	56.5	38.1	52.8	50.2
Fast-RCNN [11]	70.0	77.0	78.1	69.3	59.4	38.3	81.6	78.6	86.7	42.8	78.8	68.9	84.7	82.0	76.6	69.9	31.8	70.1	74.8	80.4	70.4
Faster-RCNN [12]	76.4	79.8	80.7	76.2	68.3	**55.9**	85.1	85.3	**89.8**	56.7	**87.8**	69.4	**88.3**	**88.9**	80.9	78.4	41.7	78.6	79.8	85.3	72.0
YOLO [15]	57.9	77.0	67.2	57.7	38.3	22.7	68.3	55.9	81.4	36.2	60.8	48.5	77.2	72.3	71.3	63.5	28.9	52.2	54.8	73.9	50.8
SSD300 [16]	77.2	78.8	85.3	75.7	71.5	49.1	85.7	86.4	87.8	**60.6**	82.7	**76.5**	84.9	86.7	84.0	79.2	51.3	77.5	78.7	86.7	76.2
SSD-TSEFFM300	**78.6**	**81.6**	**94.6**	**79.1**	**72.1**	50.2	**86.4**	**86.9**	89.1	60.3	85.6	75.7	85.6	88.3.	**84.1**	**79.6**	**54.6**	**82.1**	**80.2**	**87.1**	**79.0**
512 × 512 input																					
SSD512	79.5	84.8	85.1	81.5	73.0	57.8	87.8	88.3	87.4	**63.5**	85.4	73.2	86.2	86.7	83.9	82.5	**55.6**	81.7	79.0	**86.6**	80.0
SSD-TSEFFM512	**80.4**	**84.9**	**86.7**	**80.6**	**76.2**	**59.4**	**87.8**	**88.9**	**89.2**	61.7	**86.9**	**78.3**	**86.2**	**88.8**	**85.6**	**82.7**	55.4	**82.7**	**79.4**	84.7	**81.3**

**Table 2:** Average precision results tested on PASCAL VOC 2012 dataset (Bold values indicate the maxima).

Network	mAP	Aero	Bike	Bird	Boat	Bottle	Bus	Car	Cat	Chair	Cow	Table	Dog	Horse	Mbike	Persn	Plant	Sheep	Sofa	Train	tv
**300 × 300 input**																					
Faster-RCNN [12]	73.8	86.5	81.6	**77.2**	58.0	51.0	78.6	76.6	**93.2**	48.6	80.4	59.0	**92.1**	85.3	84.8	80.7	48.1	77.3	66.5	84.7	65.6
SSD300 [16]	75.8	88.1	82.9	74.4	61.9	47.6	82.7	78.8	91.5	58.1	80.0	64.1	89.4	85.7	85.5	82.6	50.2	**79.8**	73.6	86.6	72.1
DSSD321 [18]	76.3	87.3	83.3	75.4	64.6	46.8	82.7	76.5	92.9	59.5	78.3	64.3	91.5	86.6	86.6	82.1	**53.3**	79.6	**75.7**	85.2	**73.9**
DF-SSD300 [20]	76.5	**89.5**	85.6	72.6	**65.8**	**51.3**	82.9	79.9	92.2	**62.4**	77.5	64.5	89.5	85.4	86.4	**85.7**	51.9	77.8	72.6	85.1	71.6
SSD-TSEFFM300	**77.1**	88.6	**85.9**	76.0	65.4	46.2	**84.0**	**79.9**	92.7	58.6	**81.9**	**65.3**	91.5	**87.8**	**88.8**	82.9	52.6	79.1	75.4	**87.1**	73.8
**512 × 512 input**																					
SSD512	78.5	90.0	85.3	77.7	64.3	58.5	85.1	84.3	92.6	61.3	83.4	**65.1**	89.9	88.5	88.2	85.5	54.4	82.4	70.7	87.1	75.6
SSD-TSEFFM512	**80.2**	**90.1**	**88.2**	**81.5**	**68.4**	**59.1**	**85.6**	**85.5**	**93.7**	**63.0**	**86.1**	64.0	**90.9**	**88.6**	**89.1**	**86.4**	**59.2**	**85.9**	**73.3**	**87.8**	**75.9**

**Table 3:** TFM application results tested on PASCAL VOC 2007 dataset (Bold values indicate maxima).

Network	mAP	Conv4_3	Conv7	Conv8_2	Conv9_2	Conv10_2	Conv11_2
Original SSD	77.2						
SSD-TSEFFM	77.5	***V***					
SSD-TSEFFM	78.6	***V***	***V***				
SSD-TSEFFM	**78.7**	***V***	***V***	***V***			
SSD-TSEFFM	**78.7**	***V***	***V***	***V***	***V***		
SSD-TSEFFM	77.7	***V***	***V***	***V***	***V***	***V***	
SSD-TSEFFM	77.8	***V***	***V***	***V***	***V***	***V***	***V***
